# Prevalence of Group A Streptococcal Pharyngitis and Antibiotic Susceptibility in Paediatric Patients With Sore Throats in Gaborone, Botswana

**DOI:** 10.1111/tmi.70055

**Published:** 2025-10-16

**Authors:** Julius Chacha Mwita, Souda Sajini, Kélin Engel, Tichaona Bernard Machiya, Mark E. Engel

**Affiliations:** ^1^ Department of Internal Medicine University of Botswana Gaborone Botswana; ^2^ Department of Internal Medicine Princess Marina Hospital Gaborone Botswana; ^3^ Department of Pathology, Faculty of Medicine University of Botswana Gaborone Botswana; ^4^ Department of Medicine, Faculty of Health Sciences Cape Heart Institute, University of Cape Town Cape Town South Africa; ^5^ Department of Laboratory Services Princess Marina Hospital Gaborone Botswana; ^6^ Cochrane South Africa Cape Town South Africa; ^7^ South African Medical Research Council Cape Town South Africa

**Keywords:** antibiotic sensitivity, group a β‐haemolytic streptococcus, pharyngitis, prevalence, *Streptococcus pyogenes*

## Abstract

**Background:**

Group A β‐haemolytic Streptococcus, also known as *Streptococcus pyogenes* , commonly causes childhood pharyngitis and can lead to severe complications like acute rheumatic fever and rheumatic heart disease. Timely penicillin treatment is vital in preventing these issues. However, data on the prevalence of Group A β‐haemolytic Streptococcus pharyngitis in Botswana are limited, and there is no national surveillance for Group A β‐haemolytic Streptococcus infections.

**Objectives:**

This study aimed to determine the prevalence of Group A β‐haemolytic Streptococcus pharyngitis among children aged 8 and 18 years presenting with sore throats in selected Gaborone clinics and assess the antibiotic sensitivity patterns of Group A β‐haemolytic Streptococcus isolates.

**Methods:**

A cross‐sectional study was conducted among children aged 8–18 years suspected of pharyngitis at Nkoyaphiri and Mafitlhakgosi clinics in Gaborone. Participants were selected based on a modified Centor score of 2 or higher. Throat swabs were processed for culture, identification, and antibiotic sensitivity testing.

**Results:**

The prevalence of Group A β‐haemolytic Streptococcus pharyngitis was 7.5% (24/322; 95% CI: 0.50%–0.11%). The mean age of the participants was approximately 12 years. Group A β‐haemolytic Streptococcus isolates remained fully susceptible to penicillin, the treatment of choice, but concerns about macrolide resistance were observed in some strains.

**Conclusions:**

Group A streptococcal pharyngitis was found in 7.5% of children with sore throats in Gaborone, Botswana. All Group A β‐haemolytic Streptococcus strains were susceptible to penicillin, affirming its continued use as the preferred treatment. These results emphasize the need for accurate diagnosis and targeted antibiotic therapy to prevent complications like acute rheumatic fever and rheumatic heart disease.

## 1 Introduction

Group A β‐haemolytic streptococcal (GAS) pharyngitis is the most common bacterial cause of acute pharyngitis, especially in children. It causes 5%–15% of sore throats in adults and 20%–30% in children, resulting in over 600 million cases worldwide each year [1, 2, 3]. It can result in complications such as otitis media, sinusitis, and acute rheumatic fever. While rare in wealthy countries, these complications significantly impact health and contribute to premature death in resource‐limited regions, particularly in sub‐Saharan Africa [4]. Although streptococcal pharyngitis is self‐limiting, antibiotics are essential to prevent complications, particularly acute rheumatic fever and heart disease (RHD). Administering penicillin within 9 days of the onset of pharyngitis can effectively prevent up to 70% of acute rheumatic fever (ARF) cases, reduce the duration and severity of symptoms, and decrease the risk of transmission [5]. Therefore, accurate diagnosis and treatment of GAS infections is crucial for the primary prevention of acute rheumatic fever and heart disease.

Additionally, identifying individuals with GAS pharyngitis who require antibiotic treatment from those with viral pharyngitis reduces unnecessary antibiotic use and minimizes the risk of antimicrobial resistance [6]. Fortunately, most GAS isolates have remained susceptible to penicillin [7]. Oral erythromycin or cephalosporin is recommended for patients allergic to, as GAS isolates have also shown susceptibility to cephalosporins. Nonetheless, approximately 4%–5% of *S. pyogenes* strains are known to show resistance to macrolides [8, 9]. Data on the prevalence of GAS pharyngitis in developing countries, including Botswana, is limited compared to industrialized nations. According to Tapia et al., the prevalence of sore throats among schoolchildren aged 5 to 16 in Bamako is 25.5% [10]. The reported prevalence of sore throats among children in South Africa is 21.6% [11]. In Botswana, GAS infections are not reported to the Ministry of Health, so no surveillance program exists. Systematic data collection is essential for an effective disease control program to reduce the burden of GAS diseases in developing countries [12]. This study aimed to determine the proportion of GAS pharyngitis and describe the antibiotic sensitivity patterns of pharyngeal GAS isolates in children attending clinics in Gaborone.

## 2 Methods

### 2.1 Study Design and Setting

This cross‐sectional study was conducted in Gaborone, the capital city of Botswana. The research was conducted at the Nkoyaphiri and Mafitlhakgosi clinics between November 2018 and March 2020. These clinics provide primary healthcare services to the most populated areas of Greater Gaborone, specifically Mogoditshane and Tlokweng. Both places have experienced rapid population growth, high unemployment, poverty, inadequate housing, and significant inequality [13].

### 2.2 Participants

The study involved children aged 8 to 18 years suspected of having pharyngitis. We excluded children who had begun antibiotic treatment before enrolment.

### 2.3 Data Collection

Consecutive patients who were eligible and gave their consent to participate were enrolled until the required sample size was achieved. The details of the study were explained, and consent and assent forms were signed by all participants who agreed to take part. Data were collected simultaneously from two clinics using a standardised form that captured clinical, demographic, and laboratory parameters.

Participants were asked about their symptoms, which included fever, cough, sore throat, hoarseness, headache, rhinorrhoea, and chills. Children's axillary temperatures were measured, and examination of cervical lymph nodes for swelling and tenderness was conducted. The palate, tonsils, and pharynx were examined for any signs of exudate or swelling. In well‐lit conditions, children were asked to open their mouths as wide as possible. During this examination, sterile cotton swabs were used to collect throat samples from the posterior pharynx and tonsils, ensuring that contact with the cheeks, tongue, lips, or other mouth areas was avoided. Throat swabs were transported to the laboratory using Amies transport media.

### 2.4 Laboratory Procedure

Throat swabs were placed on blood agar plates enriched with 5% sheep blood and incubated at 35°C–37°C in a candle jar containing 5% CO_2_ for 24 to 48 h. Isolates of β‐haemolytic streptococci underwent Gram staining, catalase testing, bacitracin susceptibility testing, and PYR testing. Isolates showing sensitivity to bacitracin were subjected to additional testing for Lancefield grouping. A positive test for Lancefield group A antigen, conducted using the Streptex rapid latex agglutination method, confirmed a definitive diagnosis. The antibiotic susceptibility of GAS isolates was assessed using the disc diffusion method, following the guidelines of the Clinical and Laboratory Standards Institute and the European Committee on Antimicrobial Susceptibility Testing [14]. A pure GAS colony was suspended in peptone water and evenly spread on Mueller‐Hinton agar enriched with 5% sheep blood. Antibiotic discs were placed on the plates and incubated overnight at 37°C in a candle jar. The antibiotics tested included amoxicillin‐clavulanate, ampicillin, penicillin, ceftazidime, chloramphenicol, erythromycin, gentamicin, tetracycline, and trimethoprim‐sulfamethoxazole. Sensitivity and resistance were determined by measuring the zones of inhibition.

## 3 Data Analysis

Our sample size calculations were based on a 21.6% prevalence of GAS pharyngitis in children. To achieve a 5% margin of error at a 95% confidence level, we initially required 271 participants but increased the sample size by 20% to 322% to address potential contamination from throat swab collection. Data were analysed using SPSS Version 27, with summary statistics calculated for continuous variables (mean, standard deviation, median, min, max) and frequency distributions for categorical variables (gender, pharyngitis symptoms, GAS isolates, antibiotic sensitivity). We used the Student's t‐test for continuous data and Pearson's chi‐square test for categorical data.

## 4 Ethical Considerations

The study received ethical approval from the University of Botswana and the Ministry of Health. Permission was granted by the Greater Gaborone district management team and selected clinics. Parental consent was required for all participants, and written assent was obtained from every child. The throat culture results were communicated to the treating clinicians and the participants.

## 5 Results

**FIGURE 1:**
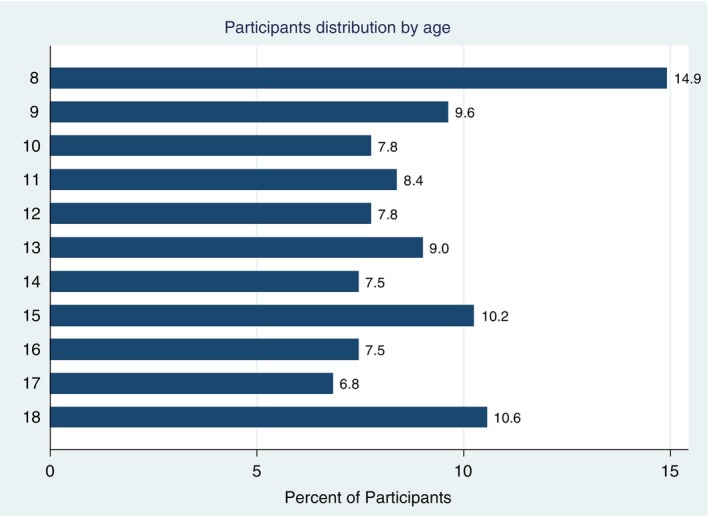
Participants' distribution by age group.

A total of 322 study participants were enrolled, with 62.1% being female. The mean age of the participants was 12.7 years, with a standard deviation of 3.4 years. The age distribution of participants is shown in Figure 1.

The participants exhibited the following symptoms: cough (60%), rhinorrhoea (56%), and hoarseness of voice (38.2%). Physical examinations revealed tonsillar swelling in 77.6% of participants, exudative throat in 40.1%, and fever in 27.3%. The median Cantor score was 2, with an interquartile range of 1 to 3.

In this study, the prevalence of GAS pharyngitis was 7.5%, with a 95% confidence interval of 5.5% to 11%. The rate was 8.0% for females (95% CI: 4.9% to 12.7%) and 6.6% for males (95% CI: 3.3% to 12.6%). Other species of Streptococcus included Group C (2.5%), Group D (1.2%), Group F (0.9%), and Group G (0.9%) (Table 1).

**TABLE 1:** Frequency of presenting complaints.

Variables	All (*n* = 322)	Throat culture		*p*
		Negative (*n* = 298)	Positive (*n* = 24)	
Rhinorrhoea—*n* (%)	148 (56)	140 (47)	8 (33.3)	0.20
Hoarseness—*n* (%)	123 (38.2)	112 (37.2)	11 (45.8)	0.42
Cough—*n* (%)	194 (60)	180 (60.4)	14 (58.3)	0.84
Tonsillar erythema—*n* (%)	139 (43.2)	121 (40.6)	18 (75)	< 0.01
Tonsillar Exudative—*n* (%)	129 (40.1)	107 (35.9)	22 (91.7)	< 0.01
Pharyngeal Exudates—*n* (%)	85 (26.4)	72 (24.2)	12 (54.2)	< 0.01
Tender cervical lymph nodes—*n* (%)	32 (9.9)	31 (10.4)	1 (4.2)	0.49
Rashes—*n* (%)	25 (7.8)	24 (8.1)	1 (4.2)	0.18
Conjunctivitis—*n* (%)	13 (4.0)	13 (4.4)	0 (0)	0.6
Tonsillar enlargement—*n* (%)	250 (77.6)	227 (76.2)	23 (95.8)	0.03
Fever—*n* (%)	88 (27.3)	83 (27.9)	5 (20.8)	0.46
Centor score, median (IQR)	2 (1–3)	2 (1–3)	3 (3–3.75)	< 0.01
Age, mean (SD), years	12.7 (3.4)	12.8 (3.4)	11.4 (3.4)	0.06

*Note:* Antimicrobial susceptibility of GAS: All 27 isolates of *Streptococcus pyogenes* exhibited sensitivity to penicillin.

## 6 Discussion

The prevalence of GAS among patients with symptomatic pharyngitis was 7.5%. All isolates were sensitive to penicillin. Our study also identified other species of Streptococcus, including Groups C, D, F, and G.

The prevalence of GAS pharyngitis in this study is lower than the pooled prevalence of 21% reported in a systematic review from a few years ago [15] and also lower than the 21.6% prevalence among children with sore throats in neighbouring South Africa [11]. Given that our recruitment method was passive, relying on participants who self‐presented at the clinics, we would have also expected to identify a significantly higher prevalence of GAS pharyngitis compared to active methods [1]. The reason for the relatively lower prevalence of GAS pharyngitis in our setting compared to other parts of Africa remains unclear. Although conducted in low‐income areas around Gaborone, Botswana's better socioeconomic conditions—considering income and development—likely explain the lower GAS burden compared to other African countries. Since most of Botswana's 2.3 million residents live within 5 km of a healthcare facility, improving access to health services significantly reduces the burden of GAS pharyngitis by enabling early diagnosis and treatment, which likely helps prevent transmission within the community [16]. The only study showing a similar prevalence of Group A Streptococcus (GAS) was conducted in Mozambique, where only 6.1% of school children with sore throats tested positive [17]. The low rate of GAS pharyngitis reported in Mozambique was linked to a study conducted outside the peak season for upper respiratory infections [17]. In contrast, our study was carried out during both peak and off‐peak seasons for these infections, suggesting that a higher prevalence might have been expected.

Furthermore, our recruitment method was passive, relying on participants who self‐presented at the clinics. Generally, passive recruitment tends to identify a significantly higher prevalence of GAS pharyngitis compared to active methods [1]. Although rare, treating sore throats caused by Group A Streptococcus with antibiotics is crucial for preventing acute rheumatic fever in this region, which has a high prevalence of RHD. Our findings indicate that approximately 7.5% of individuals with sore throats have GAS pharyngitis, a condition that requires antibiotic treatment. The other cases may be due to viral pharyngitis, which does not need medical intervention. This information is essential for effectively reducing unnecessary antibiotic use and significantly minimising the risk of antimicrobial resistance [6].

In this study, all GAS cases demonstrated complete sensitivity to penicillin. This finding strongly supports existing evidence that GAS is universally susceptible to this antibiotic, reinforcing its effectiveness in treatment [7]. Despite decades of widespread use, the reason for penicillin sensitivity remains unclear [18]. The excellent sensitivity, low cost, and minimal adverse effects make penicillin the first‐line antibiotic that most guidelines recommend [3, 19]. Considering that approximately 7.5% of individuals with sore throats may have GAS pharyngitis requiring antibiotic treatment, penicillin should be restricted to this group to prevent drug resistance, as witnessed with *Streptococcus pneumoniae* following inappropriate antibiotic use for viral infections [20].

In addition to Group A Streptococcus, beta‐haemolytic streptococci of groups C, D, F, and G were identified in the throat swabs of patients with sore throats. Group D is a gastrointestinal commensal that rarely causes pharyngitis. In contrast, β‐haemolytic Groups C and G streptococci colonise the nasopharynx, causing pharyngeal infections similar to those caused by Group A streptococcus and may lead to elevated antistreptolysin titres [21]. The low isolation rate of Groups C and G streptococci indicates that even if these organisms are associated with acute pharyngitis in paediatric patients, they represent an unusual pathogen.

This study has some limitations.

It is uncertain whether the streptococcus isolated from the pharynx is from the acute infection or a viral pharyngitis in a streptococcal carrier state. Nevertheless, current clinical practice dictates that symptomatic patients with confirmed GAS pharyngitis should be treated as though they suffer from streptococcal pharyngitis [3]. The study findings are limited to two clinics in Gaborone, so they may not be generalisable to the entire country or other regions with different healthcare access or epidemiological patterns.

In conclusion, GAS pharyngitis was identified in 7.5% of children presenting with sore throats in Gaborone, Botswana. All GAS isolates were susceptible to penicillin, supporting its continued use as the treatment of choice. These findings highlight the importance of accurate diagnosis and targeted antibiotic therapy to prevent complications such as acute rheumatic fever and rheumatic heart disease in this population.

## Conflicts of Interest

The authors declare no conflicts of interest.
